# Carbapenem-resistant *Acinetobacter* (CRAB) and outpatient antibiotic therapy (OPAT): between a rock and hard place

**DOI:** 10.1093/jacamr/dlaf201

**Published:** 2025-11-06

**Authors:** Bryan P White, Emily A Siegrist

**Affiliations:** Department of Pharmacy, OU Health, Oklahoma City, OK, USA; Department of Pharmacy, OU Health, Oklahoma City, OK, USA

*Acinetobacter baumanii-calcoaceticus* complex most commonly causes pneumonia and bacteraemia, but osteomyelitis can occur in post-surgical or trauma patients and often requires prolonged treatments of 6 weeks or more.^1,2^ Most of this duration is in the outpatient setting, often through outpatient parenteral antibiotic therapy (OPAT) programmes. The 2024 Infectious Diseases Society of America multi-drug resistant (MDR) guidance documents recommend sulbactam-durlobactam 2 g every 6 hours as first line for treatment of carbapenem-resistant *Acinetobacter baumanii* (CRAB). Recommended alternative regimens include high-dose ampicillin-sulbactam 9 g every 8 hours in combination with one other active agent, including polymyxin B, minocycline or cefiderocol 2 g every 8 hours.^3^ The 2022 ESCMID guidelines for MDR Gram negatives also recommend combination therapy with two *in vitro* active antibiotics for severe CRAB infections.^4^ They give preference to ampicillin-sulbactam for pneumonia and recommend against cefiderocol.^4^ Given the complexity of administration, limited stability data, and cost of these regimens, completing a 6-week course of therapy at home is challenging for both patients and OPAT programmes.

Recommended agents for the treatment of CRAB such as cefiderocol, ampicillin-sulbactam and sulbactam-durlobactam have significant limitations that all but preclude their outpatient use. Cefiderocol has stability data for 72 hours in an elastomeric ball for continuous infusion at 4°C,^5^ and stability for longer periods of time has not been evaluated. Although there have been case reports of continuous infusion cefiderocol administration in OPAT patients, the limited stability would require three deliveries per week from infusion companies compared with the standard once weekly delivery.^6^ As there is no increase in reimbursement for doing multiple deliveries, infusion companies often will not accept patients on antibiotics that require delivery more than once a week, and in our experience this is not an option for patients, particularly those who do not live in metropolitan areas.^7^ Without extended stability data or data for room temperature storage of premixed infusions, most patients on OPAT with cefiderocol would have to rely on minibag delivery systems, which do not require the drug to be premixed by an infusion. Instead the powder can be reconstituted by the patient in the home at the time of administration, allowing for longer stability and once weekly shipping. However, this requires a patient to self-administer two separate 1-g minibag infusions for a single dose, with each 1-g dose infused over 90 minutes (for a total of 2 g over 3 hours) every 8 hours. Patients with dexterity issues or inability to reconstitute and manipulate minibags may be unable to self-administer with this regimen at home, and additionally renal dose adjustments are limited to full vial sizes (1500-mg doses are not possible to give, which is the recommended dosing for creatinine clearance 30 to <60 mL/min). Given prolonged infusion time totalling 9 hours connected to an intravenous (IV) pole each day, this is difficult for patients and caregivers and limits the ability of patients to live normal lives. Similarly, there are no data available on sulbactam-durlobactam for continuous infusion and administration every 6 hours is not feasible for most patients on OPAT. There is also no stability data for sulbactam-durlobactam in elastomeric pumps. (personal communication Innoviva specialty therapeutics, 11 August 2025) Further, sulbactam-durlobactam has stability for only 24 hours under refrigeration and cannot be administered via a minibag, as it is only supplied as separate vials of sulbactam and durlobactam.^8^ Given the limitations in stability, infusion frequency and no minibag option, sulbactam-durlobactam is probably not logistically possible for OPAT patients. Similar to other agents mentioned, ampicillin/sulbactam has limited stability, with data only supporting storage for 72 hours at 5°C.^9^

With no stability data for sulbactam-durlobactam, known stability issues with ampicillin/sulbactam, limited options are available for CRAB patients who require OPAT. Teams are left with a choice between a suboptimal oral regimen of minocycline as monotherapy or a difficult to administer cefiderocol as two minibags q8 h given alone or in combination with minocycline or IV tigecycline. Previous studies have shown that patients have more satisfaction with simpler OPAT regimens than more complex regimens.^10^ If the aforementioned options are not feasible for a patient, placement in a facility that can give high-dose ampicillin/sulbactam may be one of the last options. We have had limited success in discharging patients who require costly antimicrobial regimens, such as cefiderocol or sulbactam-durlobactam, to facilities, as these patients are often not accepted by facilities in the USA due to cost.

To address an urgent need for patients to receive OPAT for treatment of CRAB, we would recommend the following:

(i) Pharmaceutical companies should evaluate drugs for prolonged stability under various temperatures, the most important being refrigeration and room temperature, and common delivery pump devices.
(ii) Payors should expanding evaluate reimbursement strategies similar to other long-acting antimicrobials, home health delivery restrictions and consideration for treatment outside a hospital setting.
(iii) Regulatory agencies may consider prolonging patent extensions for stability data to further encourage and permit use in a non-traditional setting.
